# Influence of provider recommendations to restart vaccines after childhood cancer on caregiver intention to vaccinate^1^

**DOI:** 10.1007/s11764-020-00890-y

**Published:** 2020-05-26

**Authors:** Echo L. Warner, Perla L. Vaca Lopez, Deanna Kepka, Karely Mann, Heydon K. Kaddas, Douglas Fair, Mark Fluchel, Elizabeth D. Knackstedt, Samantha T. Pannier, Laura Martel, Anne C. Kirchhoff

**Affiliations:** 1 University of Arizona Cancer Center, 3838 N Campbell Ave, Tucson, AZ 85719; 2 Cancer Control and Population Sciences, Huntsman Cancer Institute, 2000 Circle of Hope, Salt Lake City, Utah 84112; 3 College of Nursing, University of Utah, 10 S 2000 E, Salt Lake City, UT 84112; 4 Department of Pediatrics, Division of Pediatric Hematology/Oncology, University of Utah, 295 Chipeta Way, Salt Lake City, UT 84108; 5 Primary Children’s Hospital, Intermountain Healthcare, 100 Mario Capecchi Dr, Salt Lake City, UT 84113; 6 Department of Pediatrics, Division of Pediatric Infectious Diseases, University of Utah, 295 Chipeta Way, Salt Lake City, UT 84108; 7 Utah AIDS Education and Training Center, School of Medicine, University of Utah, 30 N 1990 E, Salt Lake City, UT 84132

**Keywords:** immunization, childhood, survivorship care, provider recommendation, caregiver

## Abstract

**Purpose:**

We studied the influence of oncology and primary care provider (PCP) recommendations on caregiver intentions to restart vaccines (e.g., catch-up or boosters) after cancer treatment.

**Methods:**

We surveyed primary caregivers ages 18 or older with a child who had completed cancer treatment 3–36 months prior (N=145) about demographics, child’s vaccination status, and healthcare factors (e.g., provider recommendations, barriers, preferences for vaccination). We compared these factors by caregiver’s intention to restart vaccines (“vaccine intention” vs. “no intent to vaccinate”) using bivariate and multivariable analyses.

**Results:**

Caregivers were primarily ages 30–39 years (54.9%), mothers (80.6%), college graduates (44.4%), Non-Hispanic (89.2%), and married (88.2%). Overall, 34.5% of caregivers did not know which vaccines their child needed. However, 65.5% of caregivers reported vaccine intention. Fewer caregivers with no intention to vaccinate believed vaccinating their child helps protect others (85.4% vs. 99.0%, p<0.01), that vaccines are needed when diseases are rare (83.7% vs. 100.0%, p<0.01), and that vaccines are safe (80.4% vs. 92.6%, p=0.03) and effective (91.5% vs. 98.9%, p=0.04) compared to vaccine intention caregivers, respectively. Provider recommendations increased caregivers’ likelihood of vaccine intention (oncologist: RR=1.65, 95% CI 1.27–2.12, p<0.01; PCP: RR=1.51, 95% CI 1.19–1.94, p<0.01).

**Conclusions:**

Provider recommendations positively influence caregivers’ intention to restart vaccines after childhood cancer. Guidelines are needed to support providers in making tailored vaccine recommendations.

**Implications for Cancer Survivors:**

Timely vaccination after childhood cancer protects patients against vaccine-preventable diseases during survivorship. Caregivers may benefit from discussing restarting vaccinations after cancer with healthcare providers.

## Introduction

Childhood immunization is one of the greatest public health advancements in the last century. Overall, 31% of pediatric cancers occur in children under age five, an age range during which the majority of childhood vaccines are recommended [1]. Recommendations for vaccination continue throughout adolescence and teenage years [1]. Guidelines generally suggest that most children with cancer begin to receive routine or catch-up vaccines once immunological recovery is complete, approximately 3–6 months following the completion of chemotherapy depending on the type of treatment administered, immunological recovery, and type of vaccine (attenuated vs. non-attenuated) [2–4]. Recent research suggests that among survivors who have not undergone bone marrow transplantation, receipt of attenuated vaccines as early as three months after treatment might result in protective antibody responses [5]. However, this research is based on small samples of patients in a limited age range. The lack of consensus regarding the optimal approach to and timing of vaccination after childhood cancer treatment remains a challenge for caregivers and clinicians alike.

While studies suggest that immunity typically can be restored successfully after cancer treatment [5–7], caregivers of childhood cancer survivors may be skeptical about the safety of vaccines for their child [8]. At the same time, some childhood cancer survivors may not see a primary care provider (PCP) in the initial years after their cancer treatment ends [3, 9], which may interrupt their access to vaccinations. Yet, there have been few studies on immunization practices of childhood cancer survivors. There is evidence that adult survivors of childhood cancer forego receipt of the yearly influenza vaccine [10], and a growing number of studies report survivors have a low uptake of the human papillomavirus (HPV) vaccine [11–13]. This limited evidence is concerning as survivors’ compromised immune systems during, and potentially after, treatment puts them at risk for contracting vaccine-preventable infections, including those that can increase cancer risk, such as HPV and Hepatitis B [14].

An emphatic recommendation from a healthcare provider is an important factor in improving vaccine intentions among caregivers in the general population [15, 16]. Whether this holds true for caregivers of children who have undergone cancer treatment remains unknown. In this study, we report findings from a clinic-based survey of primary caregivers of childhood cancer survivors. Caregivers were surveyed about their intention to restart vaccines after their child’s cancer treatment, their vaccine beliefs, and their vaccine experiences. Our goal was to determine whether intent to restart vaccines after treatment was greater among caregivers who had received a recommendation about vaccinations after cancer from their child’s provider. A secondary goal of this study was to explore caregivers’ hesitancy and barriers to restarting vaccines.

## Methods

This study was part of a larger project on vaccination after pediatric cancer at Intermountain Healthcare’s Primary Children’s Hospital (PCH). PCH is part of the Children’s Oncology Group and is the only pediatric oncology clinic in Utah, providing oncology care to the five state Mountain West region (Utah, Nevada, Idaho, Wyoming, and Montana). The University of Utah and Intermountain Healthcare’s institutional review boards approved this study. The data are available from the corresponding author upon reasonable request.

### Participants

Eligible participants included English-speaking caregivers ages 18 and older. Eligible caregivers had a child diagnosed with cancer at ages 17 or younger and had completed cancer treatment at PCH between 3–36 months prior. This timeframe was selected because most survivors return to oncology clinics for regular follow-up care during this period. We identified eligible patients through chart review.

### Procedures

Participants could complete the survey one of two ways. Caregivers whose child had a scheduled appointment were approached in person at PCH from October 2017-December 2018, or were mailed/emailed a survey if they did not have a clinic visit scheduled in this timeframe. At this point, eligible caregivers completed the informed consent process and were enrolled in the study. To minimize misinterpretation of survey questions, a member of the research team was available either in-person or over the phone to guide caregivers in completing the survey and an information sheet with vaccine names and definitions was provided.

Survey data was collected and stored electronically in REDCap; hard copies were input into REDCap. Participants received a $20 gift card.

### Survey Design

The survey was designed based on a literature review and input from experts in childhood vaccinations, including a pediatric infectious disease specialist and two pediatric oncologists (one with a focus in cancer survivorship). We pilot tested the survey with seven caregivers of pediatric cancer survivors in a focus group. Prior to the focus group, caregivers received the survey then discussed their notes and questions about the survey as a group. Using both the provider and focus group feedback, we revised the survey and obtained expert feedback from a health educator, pediatric oncologists, and PCPs to finalize the items. The final survey included 65-items with the following domains: vaccine experiences before and after cancer, vaccine preferences, caregiver demographics, childhood cancer survivor demographics, and cancer factors. Before completing the survey, caregivers were provided terms that included a list of all recommended vaccines, including the yearly influenza vaccine, and the recommended age for each vaccine, and definitions of other terms (i.e., cancer treatment, catch-up vaccines, booster vaccine, titer testing, and primary care provider). Results from questions on HPV vaccination are reported elsewhere [17].

### Survey Domains

#### Intention to restart vaccinations outcome

Our primary outcome was intention to restart vaccinations after cancer (“vaccine intention” vs. “no intent to vaccinate”. We created this outcome using two survey questions: “Has your child received any vaccinations *after* completing cancer treatment?” and “Do you plan on having your child receive vaccines they missed during cancer treatment?”

Caregivers were categorized as “vaccine intention” if they planned on having their child receive vaccines they missed during cancer treatment and/or their child had already restarted getting vaccines. Caregivers were categorized as “no intent to vaccinate” if they did not plan on having their child receive vaccines missed during treatment, if they did not know whether their child needed catch-up/booster vaccines, or if they indicated that their child did not need catch up/booster vaccines. We re-categorized eight participants to “restart vaccines” who responded that their child did not need catch up/booster vaccines because their child had received at least one vaccine after completing cancer treatment in a separate question.

### Other Measures

#### Caregiver demographics

Caregiver demographics included self-report for age, relationship with the survivor, gender, education, race, ethnicity, annual household income, insurance status, and marital status. We defined rurality of residence using Rural Urban Commuting Area codes, which are based on population density, commuting time, and urbanization in U.S. census tracts [18].

#### Childhood cancer survivors’ demographics and cancer factors

Survivor demographic factors included child’s current age, gender, race, ethnicity, insurance status, age at diagnosis, time since diagnosis, and diagnosis.

#### Healthcare and vaccination factors

Caregivers indicated whether they had received a provider recommendation for catch-up/booster vaccines from their child’s oncologist/cancer care team (yes/no) or from a PCP (yes/no). Participants also reported whether their oncologist/cancer care team had discussed when to restart their child’s vaccine schedule (yes/no/my child did not need catch-up) and whether their child has a PCP (yes/no/don’t know) and whether their child had visited a PCP since completing treatment (yes/no). Finally, participants indicated perceived barriers to completing vaccinations after cancer treatment (9 items about distance, time, transportation, scheduling, cost, knowledge) and their vaccine preferences (11 items about vaccine importance, safety, efficacy, immunity, provider discussions about vaccines, side-effects). Barriers to completing vaccinations included an open-ended “Other” item.

### Statistical analysis

We calculated summary statistics for caregiver demographic and their child’s demographic and cancer factors, and compared these factors by intention to restart vaccinations using chi-square tests and Fisher exact tests (for variables with cell sizes n≤5). A similar approach was used for the healthcare and vaccination factors. Then, to investigate factors associated with intention to restart vaccines, we fit two generalized linear models to estimate relative risks (RR) and assess the relationship of a provider recommendation from either an oncologist/cancer care team (Model 1) or a PCP (Model 2) with caregiver’s intention to restart vaccines. We chose to fit separate models for oncologists and PCPs because many pediatric oncology patients do not immediately return to primary care settings upon completion of their cancer treatment, and thus may not have had an opportunity to see a PCP or receive their recommendation for restarting vaccinations.

We applied a stepwise forward variable selection to build the final regression models, including only variables that demonstrated significant contributions to explaining variation in vaccine intentions, which included child’s current age and time since diagnosis. For the regression analyses, we performed sensitivity analyses removing n=33 caregivers who indicated that their child did not need catch up or booster vaccines. We also modeled the regressions without caregivers of patients who had received bone marrow transplant (BMT, n=8). Last, we separately ran the regressions excluding caregivers (n=20) whose child had not seen a PCP since their cancer diagnosis, but this did not appreciably change the effect estimates and these results are not shown. Missing values were excluded from the analyses. Statistical analyses were performed in Stata 14.2 and statistical significance was set at p<0.05.

For “Other” write-in responses related to barriers to vaccination and changes in vaccine beliefs after cancer, we categorized these into common areas. Relevant quotes were extracted to describe additional context to these topics. We compared open-ended responses about barriers to completing vaccinations and changes in vaccine beliefs among caregivers with vaccine intentions and those with no intent to vaccinate.

## Results

### Caregiver demographics and vaccinations after cancer

Of 196 caregivers approached in clinic, 143 were consented and completed self-administered surveys (participation rate=72.9%). We also emailed or mailed surveys to caregivers who were unable to complete the survey in clinic or who did not have an appointment scheduled during the study timeframe. Of the 50 caregivers mailed a survey, 10 completed surveys for a mail participation rate of 20%. Of the 153 completed surveys, we later found that 8 participants were ineligible, which left 145 completed surveys for analysis. Among the analytic sample, 65.5% reported intention to restart vaccines, whereas 34.4% did not (Table 1). The majority of caregivers were ages 30–39 years (54.9%), mothers (80.6%), college graduates (44.4%), married (88.2%), and Non-Hispanic (89.2%). Most caregivers reported annual household incomes of less than $79,999 (56%). The majority had health insurance (93.7%) and lived in urban locations (81.2%).

Of caregivers, 50.3% reported their child had received at least one vaccination after cancer treatment (not shown in table). Among those who said no, 9.9% reported that they did not plan on having their child receive vaccines they missed during cancer treatment, 16.9% stated they did not know if their child required vaccines, 35.2% reported that their child did not need any vaccines, and 38.0% stated that they did plan on having their child vaccinated. In Table 1, caregivers reporting vaccine intention tended to be younger compared to those with no intent to vaccinate (p=0.03).

### Cancer survivor demographics

In Table 2, most survivors were currently 5–9 years old (35.9%), male (51.0%), Non-Hispanic (85.7%), and insured (97.9%). Over one-third of survivors were diagnosed ages 0–4 years (36.6%), between 3 months to <1 year previously (53.8%), and with leukemia (37.2%). There was a higher proportion of caregivers with children ages 15–20 years at the time of survey, with no vaccine intention (36.0%) compared to those with intent to vaccinate (13.7%, p=0.01). More caregivers with intent to vaccinate had a child ages 0–4 years at diagnosis (47.4%) compared to those with no vaccine intention (16.0%, p<0.01), and a higher proportion of caregivers with vaccine intention were more than a year from diagnosis (p=0.03).

### Healthcare provider recommendation for catch-up/booster vaccines

In Table 3, caregivers who received a recommendation from an oncologist/cancer care team (62.0% vs. 38.0%, p<0.01) or PCP (52.3% vs. 47.7%, p<0.01) were more likely to report vaccine intention than those who had not received a recommendation. Likewise, 65.2% of caregivers with vaccine intention had discussed when to restart a vaccine schedule with their oncologist/cancer care team compared to only 17.0% of caregivers with no intent to vaccinate (p<0.01).

### Barriers to restarting vaccination after cancer

The most common barrier for caregivers restarting vaccines was not knowing which vaccines their child needed - and this was the case for caregivers with vaccine intention (31.6%) and those with no intent to vaccinate (40.0%, p=0.31, Table 3). Less than 10% of caregivers in either group reported barriers related to distance, time, scheduling, transportation, and cost.

For those who reported “Other” barriers (n=29), we organized write-in responses into five categories: 1) concerns about safety/side effects/causing child pain, 2) concerns about vaccines causing cancer, 3) concerns about child’s immune system not being strong enough for vaccines after treatment, 4) unclear whether their child needs vaccinations, and 5) needing information about which vaccines to get. Parents reporting vaccine intention were most commonly concerned about their child’s immune system. One caregiver commented, *“I am a little nervous getting vaccines after chemo wiped his body out.”* Caregivers reporting no intent to vaccinate commonly worried about safety/side effects. Some perceived a link between vaccines and cancer, saying, *“I feel as though vaccines may have been [a] partial cause of my son’s brain cancer.”* Multiple caregivers wanted to restart vaccinations, but needed more information, including one caregiver who said, *“We don’t always understand everything about the vaccines…I wish there was more information prior to vaccine day.”*

### Vaccine preferences

Caregivers were asked about their general preferences regarding vaccination. More caregivers reporting vaccine intention believed that vaccinating their children helps protect others (99.0% vs. 85.4%, p<0.01), and that vaccines are needed even when diseases are rare (100.0% vs. 83.7%, p<0.01) compared to those with intent to vaccinate. More caregivers reporting vaccine intention felt that vaccines are safe (92.6% vs. 80.4%, p=0.03) and effective (98.9% vs. 91.5%, p=0.04) compared to those with no intent to vaccinate. There were no other statistically significant differences by intention to restart vaccinations for other vaccine preferences. However, we did see differences in experiences when we examined write-in responses from the 22 caregivers who reported their views on vaccines had changed since their child’s diagnosis (n=2 participants skipped the write-in response). Caregivers were split, with n=13 reporting they were more supportive of vaccination after diagnosis and n=9 reporting they were less supportive of vaccination after diagnosis. Caregivers who were supportive made comments like *“I used to be [against] some [vaccines] but now I agree with them” and “[Vaccines] are so important for people around immune deficiency kids to be vaccinated so they aren’t spreading diseases.”* In contrast, caregivers who were less supportive of vaccinations after their child’s diagnosis commented *“We are much more cautious, we [worry] about so many chemicals that have been in the body”, “I worry that they [vaccines] change the body in a negative way and could have been part of the cause of cancer”*, and *“Just more aware of what is administered to my child. Now I rethink and research much more then I used to. I’m on the fence about vaccinations.”*

### Factors associated with intention to vaccinate

In multivariable models (Table 4), caregivers who received a recommendation for catch-up or booster vaccines from their oncologist/cancer care team were more likely report vaccine intention than those without a recommendation (Model 1: RR=1.65, 95%CI 1.27–2.12, p<0.01). Caregivers who had received a recommendation from a PCP were more likely to report vaccine intention than those who had not (Model 2: RR=1.51, 95%CI 1.19–1.94, p<0.01). Vaccine intention decreased with increasing child’s age for both models (Model 1: RR=0.97, 95%CI 0.95–0.99, p=0.01; Model 2: RR=0.97 95%CI 0.94–1.00, p=0.04). Greater time since diagnosis was positively associated with vaccine intention (Model 1: RR=1.22, 95%CI 1.04–1.44, p=0.02; Model 2: RR=1.24, 95%CI 1.04–1.46, p=0.01).

We performed several sensitivity analyses. First, we re-ran models removing caregivers who believed their child did not need catch up/booster vaccines. In these models, provider recommendations were still strongly associated with vaccine intention (Oncology: RR 1.35, 95%CI 1.06–1.73, p=0.01; PCP: RR=1.28, 95%CI 1.01–1.61, p=0.04). However, the influence of child’s age was no longer significant in either model.

As recommendations about timing for vaccines after a BMT differ, we did additional analyses to investigate differences with these caregivers. Although there were only 8 patients receiving a BMT in our sample, 85.7% of these caregivers had discussed when to restart their child’s vaccinations with a transplant provider. While 50% were concerned about the safety of vaccines for their child after their BMT treatment, 75% had received an oncology provider recommendation to restart vaccines and 75% intended to do so. We re-ran the regression analyses removing caregivers of BMT survivors and the effect estimates were similar (Oncology: RR: 1.75 95%CI 1.34–2.28, p<0.001 PCP: RR 1.51 95%CI 1.17–1.94, p=0.001). Time since diagnosis was not significant.

## Discussion

Timely vaccination is essential for protecting childhood cancer survivors from vaccine-preventable diseases. Over one-third of caregivers in our sample did not intend to restart their child’s vaccinations after cancer treatment. Caregivers who had discussed vaccines with a provider - either in oncology or primary care - were approximately 50% more likely to report vaccine intention after childhood cancer treatment. Clinical interventions to convey strong provider recommendations are needed to improve survivors’ receipt of immunizations after cancer treatment.

Regardless of their vaccine intention, 34.5% of all caregivers felt uncertain about which vaccines their child needed. Caregivers need guidance regarding vaccines after cancer regardless of their child’s current vaccination status. While some survivors may require catch-up or booster vaccines, others may simply benefit from a provider recommendation to continue receiving age appropriate vaccination after treatment. This is particularly relevant for caregivers of younger survivors, given that most childhood vaccines are administered under age five [1].

Some caregivers exhibited the same resistance to vaccines that is commonly found in the general public, such as worries about vaccine safety [19, 20]. A few caregivers expressed unique concerns that vaccinations may have caused their child’s cancer. These concerns demonstrate that caregivers may benefit from direct education on the safety and efficacy of vaccines after cancer treatment. There are resources for information from reputable sources, like the Centers for Disease Control and the American Academy of Pediatrics [21–23], and guidelines for immunocompromised patients [24], that clinicians can use to guide these conversations.

We also found that certain caregivers worried that their child’s immune system was not yet strong enough to develop immunity to vaccine-preventable diseases. Among those reporting no intent to vaccinate, the majority (68%) were less than one year from diagnosis compared to only 46.3% of those reporting vaccine intention. In our multivariable models, greater time from diagnosis was associated with vaccine intention, potentially because concerns about vaccinating in the initial months after treatment may subside over time. We were unable to investigate other factors such as length of cancer treatment, child’s vaccination status prior to diagnosis, and type of treatment, which may complicate providers’ vaccine recommendations. It is also possible that caregivers experience with cancer could influence personal vaccination beliefs. However, we found no differences in intention in our analyses (Table 3, p=0.65), but this should be confirmed with larger samples. In particular, caregivers’ worries about the safety of vaccines after cancer should be considered when developing interventions in both oncology and primary care settings to recommend restarting vaccines after cancer.

Without a strong commitment from oncology clinics to take responsibility for vaccinations, or appropriate training of PCPs who care for survivors in the long-term, it is unlikely that vaccine-related educational efforts will reach caregivers of pediatric cancer survivors. However, improving communication between oncologists and PCPs using a shared decision-making model for survivorship care may help [25]. Key to this model is a co-managed transition from oncology to primary care [26]. During this transition, responsibility for preventive care, such as vaccines, is shifted from oncology back to primary care [26]. Unfortunately, this transition is complex and current models may be limited by lack of vaccine specific guidance and inadequate communication between oncology and primary care. Communication about restarting vaccines could be initiated by pediatric oncology care teams during the delivery of a survivorship care plan (SCP), an evidence-based tool used to guide the transition from oncology back to primary care settings [27, 28]. As oncologists are highly trusted providers [29,30], this may be an important setting for introducing survivor and caregiver education about timely vaccination adherence after cancer treatment before they transitioning transition back to primary care. At the same time, oncologists can use the SCP as a communication tool with a patient’s PCP to improve this transition and help to provide guidance on restarting vaccinations.

While our findings demonstrate a need for providers to discuss vaccination with pediatric cancer survivors and their caregivers, barriers to these conversations in an oncology setting may include deferral of responsibility to PCPs, lack of knowledge about which vaccines to recommend and when, and not having procedures in place for vaccinating in the oncology clinic. The ambiguity regarding which provider (oncology vs. PCP) takes responsibility for post-treatment vaccination is perpetuated by the fact that, while many childhood cancer survivors continue to see an oncologist after their cancer treatment, many are also seen contemporaneously by their PCP [31]. To ensure that caregivers of childhood cancer survivors receive appropriate vaccine recommendations and have access to childhood vaccinations, flexibility in shared decision-making between oncology and primary care is needed [26].

The generalizability of our findings may be limited by a moderate sample size recruited from a single pediatric oncology clinic. Our sample is primarily Non-Hispanic White and thus, the experiences of caregivers of other races and ethnicities may be underrepresented. Our survey was not anonymous, meaning that positive response bias about vaccine intentions may have influenced our results. Our analytical decision to classify individuals who indicated that their child did not need catch-up/booster vaccines as no intent to vaccinate could have led to outcome misclassification bias. However, in sensitivity analyses, when we removed these caregivers from the regression models, both oncology and PCP recommendations remained very influential. Despite our efforts to reduce misinterpretation, the question used to measure vaccine intention may have surpassed health literacy levels for some participants. We also did not measure how the timing and strength of provider recommendations influences caregiver intentions, both of which have been shown to influence caregiver vaccine intentions in the general population. Caregiver vaccine intentions after childhood cancer treatment likely differs by vaccine type. For example, while we did not account for differences in caregiver’s intentions by vaccine type, an earlier report of HPV vaccine intentions showed that one-third of caregivers of childhood cancer who were age-eligible for the HPV vaccine were unlikely-very unlikely to get the HPV vaccine for their child [32]. Finally, there was a significantly lower proportion of rural patients with caregivers in the study (18.7%) compared to non-participants (18.7%, p=0.02). Thus, vaccine barriers for rural caregivers (e.g., travel time, distance) may be underrepresented.

In summary, provider recommendations, from either an oncologist/cancer care team member or a PCP, highly influence a caregiver’s vaccine intentions after childhood cancer treatment. High quality follow-up care for childhood cancer survivors includes childhood vaccinations, but there are no clear guidelines about how to best deliver a provider recommendation for restarting vaccinations after cancer, under what conditions caregivers are most receptive to vaccine recommendations (oncology vs. primary care), and the strengths and limitations of oncology clinics for providing education on vaccines after cancer treatment. These findings can guide future clinical interventions that test the influence of a coordinated oncology and PCP approach vaccine recommendations after cancer using a SCP to educate caregivers and survivors about needed vaccines.

## Figures and Tables

**TABLE 1:** Primary caregiver demographic characteristics by vaccine intention for their child with cancer (N=145)

	Total N=145		Vaccine intention (65.5%, n=95)		No intent to Vaccinate (34.4%, N=50)		
Caregiver	N	%	N	%	N	%	p-value^2^
**Age at survey (years)**^1^							
18–29	14	9.7	9	9.5	5	10.2	**0.03**
30–39	79	54.9	60	63.2	19	38.8	
40–49	39	27.1	19	20.0	20	40.8	
50–59	12	8.3	7	7.4	5	10.2	
**Relationship to child with cancer**^1^							0.26^3^
Mother	116	80.6	73	76.8	43	87.8	
Father	25	17.4	19	20.0	6	12.2	
Grandparent and/or Legal guardian	3	2.1	3	3.2	0	0.0	
**Gender**^1^							
Female	116	81.1	74	77.9	42	87.5	0.17
Male	27	18.9	21	22.1	6	12.5	
**Education**^1^							
High school	23	16.2	15.1	6.1	8	16.3	0.58
Some college/tech	56	39.4	34	36.6	22	44.9	
College graduate	63	44.4	44	47.3	19	38.8	
**Race**^1^							
White	129	90.9	85	90.4	44	91.7	0.54^3^
Other	13	9.1	9	9.6	4	8.3	
**Ethnicity**^1^							
Non-Hispanic	124	89.2	81	89.0	43	89.6	0.92
Hispanic	15	10.8	10	11.0	5	10.4	
**Annual household income**^1^							
<$20,000	10	7.2	5	5.4	5	10.9	0.83
$20,000-$39,999	20	14.5	13	14.1	7	15.2	
$40,000-$59,999	26	18.8	17	18.5	9	19.6	
$60,000-$79,999	22	15.9	15	16.3	7	15.2	
$80,000-$99,999	21	15.2	16	17.4	5	10.9	
>$100,000	39	28.3	26	28.3	13	28.3	
**Insurance status**^1^							
Insured	133	93.7	90	95.7	43	89.6	0.14^3^
Uninsured	9	6.3	4	4.3	5	10.4	
**Marital status**^1^							
Married/Living as married	127	88.2	87	91.6	40	81.6	0.08
Divorced/Separated/Never married	17	11.8	8	8.4	9	18.4	
**Rurality of Residence**^1^							
Urban	117	81.2	81	85.3	36	73.5	0.08
Rural	27	18.8	14	14.7	13	26.5	

1 Missing for, variable (n): age (1), relation (1), gender (2), education (3), race (3), ethnicity (6), income (7), insurance (3), marital status (1), rurality (1)

2 Chi Square test comparing proportions between caregivers reporting vaccine intention and those reporting no intent to vaccinate. Bold indicates significance at p<0.05.

3 Fisher Exact test comparing proportions between caregivers reporting vaccine intention and those reporting no intent to vaccinate. Bold indicates significance at p<0.05.

**TABLE 2:** Pediatric cancer survivor demographic characteristics by primary caregivers’ vaccine intention for their child with cancer (N=145)

	Total N=145		Vaccine intention (65.5%, n=95)		No intent to Vaccinate (34.4%, N=50)		
	N	%	N	%	N	%	p-value^2^
**Age at survey (years)**							
0–4	25	17.2	20	21.0	5	10.0	**0.01**
5–9	52	35.9	37	39.0	15	30.0	
10–14	37	25.5	25	26.3	12	24.0	
15–20	31	21.4	13	13.7	18	36.0	
**Gender**							
Female	71	49.0	52	54.7	19	38.0	0.05
Male	74	51.0	43	45.3	31	62.0	
**Race**^1^							
White	129	89.6	85	90.4	44	88.0	0.65
Other	15	10.4	9	9.6	6	12.0	
**Ethnicity**^1^							
Non-Hispanic	120	85.7	79	85.9	41	85.4	0.94
Hispanic	20	14.3	13	14.1	7	14.6	
**Insurance status**^1^							
Insured	141	97.9	92	97.9	49	98.0	0.72^3^
Uninsured	3	2.1	2	2.1	1	2.0	
**Age at diagnosis (years)**							
0–4	53	36.6	45	47.4	8	16.0	**<0.01**
5–9	39	26.9	23	24.2	16	32.0	
10–14	33	22.8	21	22.1	12	24.0	
15–17	20	13.8	6	6.3	14	28.0	
**Time since diagnosis**							
3 months to <1 year	78	53.8	44	46.3	34	68.0	**0.03**^3^
1 to <2years	56	38.6	41	43.2	15	30.0	
2 to <3 years	11	7.6	10	10.5	1	2.0	
**Diagnosis**							
Leukemia	54	37.2	42	44.2	12	24.0	0.11
Brain/Central Nervous	21	14.5	13	13.7	8	16.0	
Lymphoma	26	17.9	14	14.7	12	24.0	
Other	44	30.3	26	27.4	18	36.0	

1 Missing for, variable (n): race (1), ethnicity (5), health insurance (1)

2 Chi Square test comparing proportions between caregivers reporting vaccine intention and those reporting no intent to vaccinate. Bold indicates significance at p<0.05.

3 Fishers Exact test comparing proportions between caregivers reporting vaccine intention and those reporting no intent to vaccinate. Bold indicates significance at p<0.05.

**TABLE 3:** Factors associated with primary caregivers’ vaccine intention for their child with cancer (N=145)

	Total N=145		Vaccine intention (65.5%, n=95)		No intent to Vaccinate (34.4%, N=50)		
	N	%	N	%	N	%	p-value^2^
**Provider recommended catch-up/booster**							
**Oncologist/cancer care team**							
Yes	66	46.8	57	62.0	9	18.4	**<0.01**
No	75	53.2	35	38.0	40	81.6	
**Primary care provider**							
Yes	51	37.8	46	52.3	5	10.6	**<0.01**
No	84	62.2	42	47.7	42	89.4	
**Oncologist/cancer care team discuss when to restart vaccine schedule**^1^							
Yes	66	48.5	58	65.2	8	17.0	**<0.01**
No	45	33.1	23	25.8	22	46.8	
My child did not need catch-up	25	18.4	8	9.0	17	36.2	
**Child has a primary care provider**							
Yes	135	93.1	89	93.7	46	92.0	0.38
No	8	5.5	4	4.2	4	8.0	
Don’t know	2	1.4	2	2.1	0	0.0	
**Primary care provider visit since completing treatment**^1^							
Yes	115	85.2	78	87.6	37	80.4	0.26
No	20	14.8	11	12.4	9	19.6	
**Barriers to completing (select all that apply)**							
Distance to clinic	8	5.5	5	5.3	3	6.0	0.56^3^
Time needed to get to clinic	6	4.1	6	6.3	0	0.0	0.08^3^
Scheduling clinic appointments	12	8.3	8	8.4	4	8.0	0.60^3^
Wait time at clinic	3	2.1	2	2.1	1	2.0	0.73^3^
Transportation costs to get to clinic	0	0.0	0	0.0	0	0.0	NA
Cost of clinic visit	3	2.1	2	2.1	1	2.0	0.73^3^
Cost of the vaccine	5	3.4	3	3.2	2	4.0	0.56^3^
Don’t know which vaccines to get	50	34.5	30	31.6	20	40.0	0.31
Other	29	20.0	17	17.9	12	24.0	0.38
**Vaccine preferences (yes response only)**							
Getting recommended vaccines is important	136	96.4	93	97.9	43	93.5	0.20^3^
Vaccinating my child helps protect others	135	94.4	94	99.0	41	85.4	**<0.01**^3^
Vaccines needed when diseases are rare	136	94.4	95	100.0	41	83.7	**<0.01**^3^
Vaccines are safe	125	88.6	88	92.6	37	80.4	**0.03**
Vaccines are effective	137	96.5	94	98.9	43	91.5	**0.04**
Children develop better immunity by getting sick	24	17.4	12	13.0	12	26.1	0.06
Comfortable discussing vaccine concerns with providers	133	93.0	90	94.7	43	89.6	0.25
Providers give enough info on vaccines/side effects	107	76.4	71	77.2	36	75.0	0.77
Some age-recommended vaccines are unnecessary	17	11.9	8	8.4	9	18.7	0.07
Doctor reluctant to administer requested vaccines	2	1.4	2	2.1	0	0.0	0.43^3^
The cancer diagnosis changed my view of vaccines	24	16.8	15	15.8	9	18.7	0.65

1 Missing for, variable (n): Oncologist/cancer care team recommended catch-up/booster (4), PCP recommended catch-up/booster (10), Oncologist/cancer care team discuss when to restart (9), PCP since completing treatment (10), getting vaccines is important (4), vaccinating my child helps protect others (2), vaccines needed when diseases are rare (1), vaccines are safe (4), vaccines are effective (3), children develop better immunity by getting sick (7), comfortable discussing vaccine concerns with providers (2), providers give enough info on vaccines/side effects (5), some age-recommended vaccines are unnecessary (2), doctor reluctant to administer requested vaccines (2), the cancer diagnosis changed my view of vaccines (2)

2 Chi Square test comparing proportions between caregivers reporting vaccine intention and those reporting no intent to vaccinate. Bold indicates significance at p<0.05.

3 Fisher Exact test comparing proportions between caregivers reporting vaccine intention and those reporting no intent to vaccinate. Bold indicates significance at p<0.05.

**TABLE 4:** Relative risks of provider recommendation for catch-up or booster vaccination with primary caregivers’ vaccine intention for their child with cancer

		Model 1			Model 2	
	RR	95%CI	p-value^1^	RR	95%CI	p-value^1^
**Oncologist/cancer care team recommendation**^2^						
Yes	**1.65**	**1.27–2.12**	**<0.01**	--	--	--
No	Ref.					
**Primary care provider recommendation**^2^						
Yes	--	--	--	**1.51**	**1.19–1.94**	**<0.01**
No				Ref.		
**Child’s current age (years)**	**0.97**	**0.95–0.99**	**0.01**	**.97**	**0.94–1.00**	**0.04**
**Time since diagnosis (years)**	**1.22**	**1.04–1.44**	**0.02**	**1.24**	**1.04–1.46**	**0.01**

1 Bold indicates significance at p<0.05.

2 Outcomes were missing for: oncologist/cancer care team (n=4), primary care provider (n=10). All listed variables included in each model.

## Abbreviations

CI: Confidence Interval
COG: Children’s Oncology Group
IDSA: Infectious Disease Society of America
HPV: Human Papillomavirus
PCH: Primary Children’s Hospital
PCP: Primary Care Provider
RR: Relative Risk
